# Somatic Functional Deletions of Upstream Open Reading Frame-Associated Initiation and Termination Codons in Human Cancer

**DOI:** 10.3390/biomedicines9060618

**Published:** 2021-05-29

**Authors:** Lara Jürgens, Felix Manske, Elvira Hubert, Tabea Kischka, Lea Flötotto, Oliver Klaas, Victoria Shabardina, Christoph Schliemann, Wojciech Makalowski, Klaus Wethmar

**Affiliations:** 1 Department of Medicine A, Hematology, Oncology, Hemostaseology and Pneumology, University Hospital Münster, 48149 Münster, Germany; Lara.Juergens@ukmuenster.de (L.J.); Elvira.Hubert@ukmuenster.de (E.H.); Lea.Floetotto@ukmuenster.de (L.F.); Oliver.Klaas@ukmuenster.de (O.K.); Christoph.Schliemann@ukmuenster.de (C.S.); 2 Faculty of Medicine, Institute of Bioinformatics, University of Münster, 48149 Münster, Germany; felix.manske@uni-muenster.de (F.M.); Tabea.kischka@gmail.com (T.K.); wojmak@uni-muenster.de (W.M.); 3 Institute of Evolutionary Biology, CSIC-Unversitat Pompeu Frabra, 08002 Barcelona, Spain; victoria.shabardina@upf.edu

**Keywords:** gene expression regulation, upstream open reading frame, cancer, whole-exome-sequencing, translational control

## Abstract

Upstream open reading frame (uORF)-mediated translational control has emerged as an important regulatory mechanism in human health and disease. However, a systematic search for cancer-associated somatic uORF mutations has not been performed. Here, we analyzed the genetic variability at canonical (uAUG) and alternative translational initiation sites (aTISs), as well as the associated upstream termination codons (uStops) in 3394 whole-exome-sequencing datasets from patient samples of breast, colon, lung, prostate, and skin cancer and of acute myeloid leukemia, provided by The Cancer Genome Atlas research network. We found that 66.5% of patient samples were affected by at least one of 5277 recurrent uORF-associated somatic single nucleotide variants altering 446 uAUG, 347 uStop, and 4733 aTIS codons. While twelve uORF variants were detected in all entities, 17 variants occurred in all five types of solid cancer analyzed here. Highest frequencies of individual somatic variants in the TLSs of NBPF20 and CHCHD2 reached 10.1% among LAML and 8.1% among skin cancer patients, respectively. Functional evaluation by dual luciferase reporter assays identified 19 uORF variants causing significant translational deregulation of the associated main coding sequence, ranging from 1.73-fold induction for an AUG.1 > UUG variant in SETD4 to 0.006-fold repression for a CUG.6 > GUG variant in HLA-DRB1. These data suggest that somatic uORF mutations are highly prevalent in human malignancies and that defective translational regulation of protein expression may contribute to the onset or progression of cancer.

## 1. Introduction

Ribosome profiling and recent mass spectrometric analyses uncovered high translational activity outside of previously annotated protein coding sequences (CDSs) [1,2]. A large fraction of such ectopic translational events mapped to upstream open reading frames (uORFs) initiated by canonical (uAUG) or alternative translational initiation sites (aTISs) [3,4,5,6]. While more than half of all human 5′-transcript leader sequences (TLSs) contain uAUG codons [6], virtually all eukaryotic TLSs contain aTISs, differing in one base from the classical AUG triplet [1,5,7,8,9].

Globally, uORFs are considered to repress downstream translation [10]. During cap-dependent translation, the 43S pre-initiation complex scans the mRNA in 5′- to 3′-direction and initiates translation at the first start codon suitable for ribosomal initiation [5,11]. The regulatory effect on the translation rates of uORF-associated CDSs is largely dependent on transcript specific features, including the length, number, position, and the peptide sequence of individual uORFs, as well as the sequence context surrounding the uORF initiation and termination codons [3,12,13]. Optimal support for translational initiation is mediated by a sequence context of GCCRCCaugG (Kozak consensus sequence), with core bases underlined and R representing a purine base [14]. Of note, recent observations in zebrafish substantially broadened the spectrum of favorable, initiation promoting sequence contexts in eukaryotes [15]. Translational initiation at a uORF always results in consumption of a functional ribosomal pre-initiation complex that will no longer be available for downstream initiation, unless reloaded with essential initiation co-factors [16]. Ribosomal initiation at uORFs may also hamper CDS translation by simple dissociation of post-termination ribosomes from the mRNA or by the induction of ribosome stalling at the associated uStop codons and the subsequent mediation of nonsense-mediated mRNA decay [17]. Translation of the CDS in uORF containing transcripts requires ribosomes to either bypass the uORF initiation codon by leaky scanning or to reinitiate at the CDS after uORF translation [5]. Due to the high variability of uORF sequences and the structural complexity of human TLSs, the regulatory impact of an individual uORF cannot be predicted from sequence analyses alone, but always requires experimental testing.

Accumulating evidence suggests a (patho-)physiological important role of uORF-mediated translational control in health and disease [2,18,19,20,21]. Upstream ORFs have been implicated in directing protein expression during cellular integrated stress responses, as translational regulation allows for immediate response to changing environmental conditions, bypassing the need for time consuming transcription of new mRNA [5,22,23,24,25]. With respect to human diseases, Marie Unna hereditary hypotrichosis is caused by defective uORF-mediated translational control [26]. Here, the loss of a uAUG in the hairless homolog gene (HR) results in enhanced translation of HR and subsequent dysfunction of hair follicles. Furthermore, cases of hereditary thrombocytosis were linked to the loss of uORF regulation in an alternatively spliced transcript [27]. Upstream ORFs are predominantly present in transcripts of proto-oncogenes and tumor suppressor genes [11,20]. Two examples have been identified, where genetic alterations of the uORF sequences in cyclin dependent kinase 2A and 1B promote the development of hereditary melanoma and malignancies resembling multiple endocrine neoplasia syndrome type 4, respectively [28,29]. Additionally, a substantial number of hereditary uORF-associated single nucleotide polymorphisms (SNPs) have been linked to various human diseases, but very few have been functionally investigated [30,31,32].

Aiming to better characterize the landscape of uORF-associated mutations in cancer, we here determined the prevalence of somatic genetic variation at canonical and alternative uORF initiation codons plus the associated termination codons in primary patient samples derived from five major types of solid cancer and in acute myeloid leukemia. Targeted analysis of several thousands of whole-exome sequencing datasets of The Cancer Genome Atlas (TCGA) revealed a high number of previously unrecognized recurrent somatic mutations at uAUG, aTIS, and uStop codons. We found that several of the identified uORF-associated variants induced deregulation of downstream CDS translation. These data suggest that genetic variation at uORF initiation and termination codons may result in impaired expression of uORF-regulated proteins and may contribute to malignant transformation and cancer progression.

## 2. Materials and Methods

### 2.1. Definition of TLSs and uORF-Associated Genomic Positions

The genomic positions of uORF-associated sequences were determined using a custom uORF-finder script as previously described [21]. The current version of the script is available at https://doi.org/10.5281/zenodo.4776480, accession date: 22 May 2021. The genomic coordinates of TLSs were based on the transcripts as defined in the “RefSeq Curated” table retrieved from the UCSC Table Browser for assembly hg38 (ftp://hgdownload.soe.ucsc.edu/goldenPath/hg38/database/ncbiRefSeqCurated.txt.gz, date of accession and download: 9 February 2020). All uORFs in all RefSeq transcript variants (TVs) were defined by canonical and near-cognate initiation codons (AUG and UUG, GUG, CUG, AAG, AGG, ACG, AUA, AUU, AUC, respectively) followed by a downstream in-frame termination codon. All three reading frames (RFs) of the TLSs were evaluated.

### 2.2. Variation Data Obtained from GDC Data Portal

Variant Call Format (VCF) files with nucleotide variation information were retrieved from the National Cancer Institute GDC Data Portal (https://portal.gdc.cancer.gov/, Data Release Number 12, accession date: 14 June 2018). We chose six types of cancer from TCGA: breast invasive carcinoma (BRCA), colon adenocarcinoma (COAD), skin cutaneous melanoma (SKCM), lung adenocarcinoma (LUAD), prostate adenocarcinoma (PRAD), and acute myeloid leukemia (LAML). For individual patient samples, the VCF files generated as output of the MuTect2 pipeline for whole-exome sequencing (WES) data were downloaded. The exact query to get all VCF files was: cases.project.program.name in [“TCGA”] and cases.project.project_id in [“TCGA-BRCA”,”TCGA-COAD”,”TCGA-LAML”, ”TCGA-LUAD”,”TCGA-PRAD”,”TCGA-SKCM”] and files.analysis.workflow_type in [“MuTect2”] and files.data_format in [“vcf”] and files.experimental_strategy in [“WXS”]. There were 1080 VCF files for BRCA, 493 for COAD, 182 for LAML, 670 for LUAD, 503 for PRAD, and 472 for SKCM, including 1044 patients for BRCA, 433 for COAD, 149 for LAML, 569 for LUAD, 498 for PRAD, and 470 for SKCM.

### 2.3. Calculation of Read Coverage

The read depths at uAUG, aTIS, and uStop positions of the WES datasets were extracted from the Binary Alignment Map (BAM) files on which the VCF files were based upon. The query used to get a list of all BAM files was: cases.project.program.name in [“TCGA”] and cases.project.project_id in [“TCGA-BRCA”,”TCGA-COAD”,”TCGA-LAML”,”TCGA-LUAD”,”TCGA-PRAD”,”TCGA-SKCM”] and files.data_format in [“bam”] and files.experimental_strategy in [“WXS”]. Due to storage size limitations, we cut BAM files to the relevant regions. We first merged the regions of the uORF exons and all exons within the 5′-UTR. Region coordinates were based on UCSC’s “NCBI RefSeq Genes” (refGene) table (ftp://hgdownload.soe.ucsc.edu/goldenPath/hg38/database/refGene.txt.gz; date of accession and download 7 February 2019). Each region was then extended by 100 nt in both directions and overlaps were again merged. Finally, BAM files were intersected with the resulting regions of interest. During our analyses we found that uORF discovery based on UCSC’s “RefSeq Curated” table (see Section 2.1) provided more accurate results. Thus, we intersected the previously extracted coverages with the positions of uStart and uStop codons in our new dataset. We only retained positions which appeared in both datasets. For each BAM file, we then calculated the fraction of genomic positions mapping to uStart and uStop codons covered by ≥10 reads using SAMTools version 1.9 [33]. All operations on the BAM files were performed using BEDTools 2.27.1 [34].

### 2.4. Identification of Somatic Variants at uAUG, aTIS, and uStop Codons

A custom script, available at https://doi.org/10.5281/zenodo.4776480 (accession date 22 May 2021) scanned the patient-derived VCF files for overlap with previously defined uORF start and stop codon positions. Detailed information on the affected uORFs and mutations was output in table format. Metadata for the mutations were derived from dbSNP 151 (date of accession and download: 2018-04-18) [35]. In order to filter spurious mutations, the program calculated a set of filters which were then applied manually.

### 2.5. Filtering of Raw Variability Data

All screened positions were filtered for their genetic variability. To enhance sensitivity of our analysis and to exclude spurious variants, common MuTect2 filters were declined and replaced by a set of custom filters. In order to qualify for further analyses a nucleotide position must have been covered by ≥10 reads in the sequencing data of both the tumor and the associated normal control sample, and had to be located within the TLS of at least one TV with a RefSeq ID. Somatic mutations were considered, when three or more reads in the tumor sample supported alternative bases and when the ratio of alternative reads and reference reads was ≥4 times higher in the tumor sample as compared to the normal control sample. All filters described here were based on the VCF files.

### 2.6. Selection of uORF-Associated Variants for Functional Testing

All recurrent somatic uORF-associated variants were manually curated for additional structural and functional features to define a set of single nucleotide variants (SNVs) suitable for experimental analysis. Initially, all variants with an average dbSNP frequency of >10^−5^ (reported by TOPMED, GnomAD, and ExAC projects) were excluded manually. Further criteria to positively select SNVs for functional testing were: Low numbers of uAUG uORFs in TLS (≤6), short distance of uStop to CDS start or uORF overlapped into CDS, high proportion of TVs shared the respective uORF, high rate of cancer-specific somatic mutations, SNV was observed in more than one type of cancer, SNV affected more than one uORF, or SNV occurred in a gene associated with tumor formation or progression. To select termination codons for experimental analysis, specific additional criteria were applied: extended uORF newly overlapped into the CDS or led to a significant increase of uORF length, uStop was related to multiple in-frame upstream initiation codons.

### 2.7. Cell Lines and Plasmids

The HEK293T cell line was obtained from ATCC and cultured in DMEM medium containing 10% fetal bovine serum (FBS) and Penicillin/Streptomycin. For uORF variant testing a representative TLS for the respective gene was selected, synthesized by GeneArt^®^ gene synthesis (Invitrogen™, Carlsbad, USA), and cloned into a translational control reporter plasmid via NheI, SmaI, BglII, XhoI, or SacII restriction sites depending on the insert (Supplementary Table S1), as previously described [6]. Full length TLSs including the complete 5′-region of the transcript, the endogenous CDS initiation codon, and the +4 Kozak base were ligated in-frame to the Firefly luciferase coding sequence. All cloning procedures were performed using standard restriction/ligation protocols, as previously described [21]. Most TLS constructs were synthesized in wild type (wt) and ∆uORF versions. Occasionally, the observed mutations were introduced by site-directed mutagenesis (SDM) following standard PCR reaction protocols using mismatching primers (Supplementary Table S2). Correct introduction of desired mutations and preservation of the surrounding complete TLS sequence were validated by Sanger sequencing for each target. The pRL-CMV vector (Promega, Madison, WI, USA) containing the Renilla luciferase sequence was used as internal control in dual luciferase reporter assays.

### 2.8. Dual Luciferase Reporter Assays

Dual luciferase reporter assays were performed as previously described [36]. Briefly, for each Firefly vector construct HEK293T cells were seeded in 24-well plates at a density of 50,000 cells/well. After 24 h of growth cells were transiently co-transfected with TLS-specific amounts of translational control reporter plasmids (wt or ∆uORF, respectively) and 75 ng of the pRL-CMV vector using Metafectene^®^ transfection reagent according to manufacturer’s protocol (Biontex Laboratories, Munich, Germany). For individual TLSs the amount of transfected Firefly luciferase vector was adjusted to the optimal linear range of the plate reader. 44 h after transfection the cells were washed with PBS and lysed with 100 μL luciferase lysis buffer [36] containing protease inhibitor cocktail, while shaking for 30 min at 4 °C. The lysates were centrifuged at 21,000× *g* and 4 °C for 10 min. Measurement of luciferase signal was performed in Luminometer Victor™ X3 (PerkinElmer, Waltham, WI, USA) using 10 μL of protein lysate in a white 96-well plate in triplicates with an automatically injector-based addition of 80 μL luciferase reaction buffer A (0.2 M Tris pH 8, 0.025 M DTT, 0.015 M MgSO_4_, 0.1 mM EDTA; pH 8 and addition of 1 mM ATP, 0.2 mM D-luciferin) and 50 μL luciferase reaction buffer B (0.5 M Na_2_SO_4_, 0.025 M Na_4_PPi, 0.5 M NaCl, 1.5 mM EDTA, 0.01 M NaAc; pH 5 and addition of 0.05 mM APMBT, 0.004 mM benzyl coelenterazine). The Firefly and Renilla catalyzed luminescent reactions were measured after subsequent addition of respective reaction buffers.

### 2.9. RNA Preparation and qRT-PCR

RNA was prepared using the NucleoSpin^®^ RNA Kit (Macherey-Nagel, Dueren, Germany) following the instructions provided by the manufacturer including DNAse I digestion of 1 μg RNA to eliminate residual DNA. cDNA was generated using the RevertAid First Strand cDNA synthesis Kit (Thermo Fisher Scientific, Waltham, WI, USA) from 200 ng RNA per reaction. SYBR Green-based quantitative PCR was performed using Luna Universal qPCR Master Mix (NEB, Ipswich, USA) and the following primers: Firefly_for ATCCATCTTGCTCCAACACC, Firefly_rev TCGCGGTTGTTACTTGACTG, Renilla_for GGAATTATAATGCTTATCTACGTGC, Renilla_rev CTTGCGAAAAATGAAGACCTTTTAC

## 3. Results

### 3.1. Read Coverage at uAUG, aTIS, and uStop Codons in TCGA-Derived Whole-Exome Sequencing Datasets

**Figure 1:**
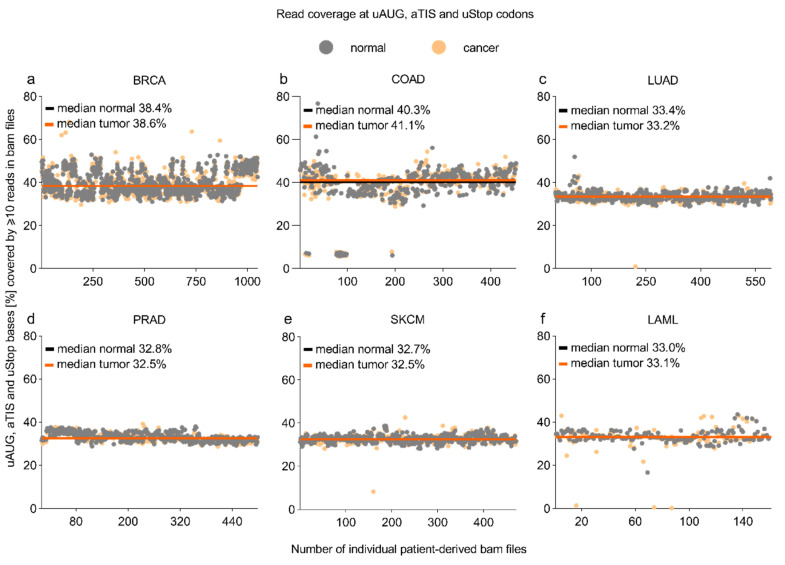
Coverage of uORF-associated uAUG, aTIS, and uStop codons in WES datasets. Dot plots indicating the proportion of genomic positions of canonical (uAUG) and alternative initiation sites (aTISs), as well as the associated termination codons (uStops) covered by ≥10 reads in tumor (orange dots) and normal control (grey dots) patient samples. The coverage analysis was performed using TCGA-derived whole-exome sequencing BAM files of (**a**) breast invasive carcinoma (BRCA), (**b**) colon adenocarcinoma (COAD), (**c**) lung adenocarcinoma (LUAD), (**d**) prostate adenocarcinoma (PRAD), (**e**) skin cutaneous melanoma (SKCM), (**f**) acute myeloid leukemia (LAML), and the related normal controls. Orange and black lines indicate the median coverage of entity-specific tumor and normal control samples, respectively.

To determine the read coverage in whole-exome sequencing (WES) datasets provided by the Cancer Genome Atlas (http://cancergenome.nih.gov, accession date: 14 June 2018), we first localized all uORF-associated canonical (uAUG) and alternative initiation sites (aTISs: UUG, GUG, CUG, AAG, AGG, ACG, AUA, AUU, AUC) plus all uORF-related upstream termination codons (uStops: UAA, UAG, UGA) in the current human genome assembly (hg38). Computational analyses identified a total of 190,878 uAUG-, 2,515,399 aTIS-, and 624,157 uStop-associated genomic nucleotide positions (Table 1). Sequencing coverage at these positions varied among the WES datasets of acute myeloid leukemia (LAML) and five major entities of solid cancer investigated here, i.e., breast invasive carcinoma (BRCA), colon adenocarcinoma (COAD), lung adenocarcinoma (LUAD), prostate adenocarcinoma (PRAD), skin cutaneous melanoma (SKCM). For individual types of cancer, the median proportion of uORF-associated positions sufficiently covered for mutational analyses ranged from 32.5% in the PRAD and SKCM cohorts to 41.1% in the COAD cohort (Figure 1, Supplementary Data S1). No significant differences were observed for tumor and control tissue-derived samples of individual entities. Limited coverage of uORF-associated positions was expected, as most exome sequencing studies focused on the coding regions of genes and neglected exons exclusively encoding 5′- and 3′-regulatory regions of the transcripts. Accordingly, we observed the highest proportion of sufficiently covered nucleotide positions for uStop codons (44.2% to 52.4%), intermediate coverage for uAUG codons (35.4% to 43.7%), and lowest coverage for aTIS codons (29.2% to 37.9%), showing a similar pattern for all cohorts analyzed here (Supplementary Figure S1).

### 3.2. Identification of Recurrent Somatic Genetic Variation at uAUG, aTIS, and uStop Codons

We then analyzed the WES datasets derived from 1044 BRCA, 433 COAD, 569 LUAD, 498 PRAD, 470 SKCM, and 149 LAML patients for genetic variation at all uORF-associated initiation and termination codons. Based on computational filtering of TCGA-derived raw variation data, we identified a total number of 48,491 unique uORF-associated single nucleotide variants (SNVs) across all six types of cancer (Table 1, Supplementary Data S2). We generated a web interface allowing interactive filtering for all uORF-associated variants identified in the current study (http://bioinformatics.uni-muenster.de:3838/uorf_result_display, accession date: 8 March 2021). The SKCM and LAML cohorts showed highest (*n* = 26,051) and lowest absolute numbers (*n* = 1913) of uORF-associated SNVs, respectively. Virtually all patients were affected by one or more uORF-related variant position. Because of the anticipated stronger relevance for tumor development and/or progression, we then applied additional filters to identify recurrent somatic SNVs, i.e., affecting ≥2 individual patients of a specific type of cancer. We identified 5277 uORF-related positions showing recurrent somatic variation, including 2362 newly identified somatic variants not previously annotated in dbSNP (https://www.ncbi.nlm.nih.gov/snp/, date of accession and download: 18 April 2018) [35]. Similar to the distribution of all uORF-associated SNVs, the highest number of recurrent somatic variants was observed for the SKCM-derived datasets (*n* = 3748), followed by BRCA (*n* = 1029), LUAD (*n* = 494), COAD (*n* = 339), LAML (*n* = 258), and PRAD (*n* = 114). Depending on the type of cancer, 38.8% (PRAD) to 86.8% (SKCM) of patients were affected by at least one recurrent somatic uORF variant. 

More stringent filtering for those somatic uORF variants that occurred in ≥1% of patients per entity revealed that still 20.9% (PRAD) to 75.2% (LAML) of patients were affected by one or more somatic uORF mutation. Among all recurrent somatic SNVs, the proportion of newly identified somatic variants without previous annotation in dbSNP ranged from 2.6% in the PRAD cohort to 56.4% in the SKCM cohort.

**Figure 2:**
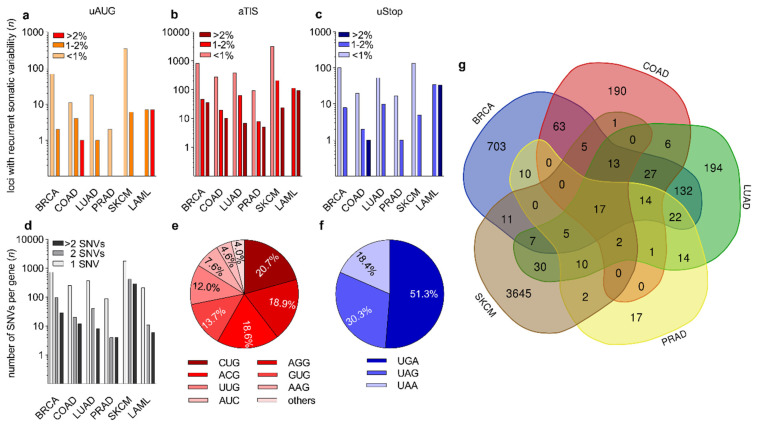
Recurrent somatic uORF-associated SNVs in six types of human cancer. (**a**–**c**) Bar graphs showing the number (*n*) of (**a**) uAUG-, (**b**) aTIS-, and (**c**) uStop-associated recurrent SNVs observed in breast invasive carcinoma (BRCA), colon adenocarcinoma (COAD), lung adenocarcinoma (LUAD), prostate adenocarcinoma (PRAD), skin cutaneous melanoma (SKCM), and acute myeloid leukemia (LAML). Coloring of bars indicates whether SNVs affected <1%, 1–2%, or >2% of patients for each type of cancer. (**d**) Bar graph showing the number of genes (*n*) affected by 1, 2, or >2 SNVs in indicated types of cancer. (**e**,**f**) Pie charts displaying the relative distribution of recurrent somatic SNVs affecting the indicated types of (**e**) aTIS and (**f**) uStop codons. (others = AUU (2.4%) and AUA (1.5%)). (**g**) Venn diagram showing the number of shared recurrent somatic SNVs among indicated types of cancer.

The relative distribution of recurrent somatic variants affecting uAUG, aTIS, or uStop was 8.5%, 89.7%, and 6.6%, respectively, with individual SNVs occasionally affecting uORF-associated initiation and termination codons concomitantly (Figure 2a–c, Supplementary Data S2). While the majority of uORF-associated SNVs occurred at low frequencies, we detected 451 SNVs affecting 1–2% and 132 SNVs affecting >2% across all entity-specific cohorts. Of note, all recurrent somatic SNVs (*n* = 258) in the LAML cohort of 149 patients had a frequency of >1%. Several SNVs in the BRCA, LAML, and SKCM cohorts affected >5% of patients (Supplementary Data S2), with the highest frequency of recurrence being observed for a somatic UUG > UUU variant in the Neuroblastoma Breakpoint Family Member 20 (NBPF20) in 10.1% of LAML patient samples. This position showed an average germline variant allele frequency (VAF) of 0.01% in TopMed, ExAC, and GnomAD cohorts (Supplementary Table S3). For the solid tumor entities, the highest frequency of recurrence was detected for an AUC > AUU variant affecting the TLS of Coiled-coil-helix-coiled-coil-helix domain-containing protein 2 (CHCHD2) in 8.1% of SKCM samples, with an average dbSNP-annotated VAF of 8 × 10^−6^. Highest frequencies of newly identified somatic SNVs without any previous dbSNP annotation were observed for a UAA > AAA variant in Follicular Dendritic Cell Secreted Protein (FDCSP) in 6.0% of LAML samples, and for an ACG > ACU variant in HMG domain-containing protein 4 (HMGXB4) in 2.5% of SKCM samples. Among all genes harboring recurrent somatic uORF variants, a substantial fraction of genes was affected at two or more uORF-associated positions. In the SKCM cohort we detected 710 of such genes (28.8% of all affected genes). In the BRCA, LUAD, COAD, LAML, and PRAD cohorts 124 (14.6%), 48 (11.4%), 32 (11.2%), 17 (7.4%), and 8 (8.2%) genes carried >1 uORF variant, respectively (Figure 2d).

The genes affected by the highest number of uORF-associated somatic variants were Cadherin 1 (CDH1) and Tumor Protein P53 Binding Protein 1 (TP53BP1), both detected in the SKCM dataset and both harboring 13 individual variants (Supplementary Data S2). The most common non-canonical uORF initiation codons altered by somatic mutations were CUG (20.7%), AGG (18.9%), ACG (18.6%), and GUG (13.7%) (Figure 2e). With respect to the uORF-associated termination codons, 51.3% of the somatic loss-of-uStop variants affected UGA codons, while 30.3% and 18.4% of variants functionally deleted UAG and UAA codons, respectively (Figure 2f). Multiple somatic variants affecting uAUG, aTIS, or uStop codons occurred in various types of cancer (Figure 2g). Twelve recurrent somatic uORF-associated variants were identified in all types of cancer analyzed here, 17 were found in all solid cancers (Supplementary Table S4), and 34 SNVs were found in four of the five solid cancer entities (Figure 2g).

### 3.3. Defective Translational Regulation by uORF-Associated Somatic SNVs

**Figure 3:**
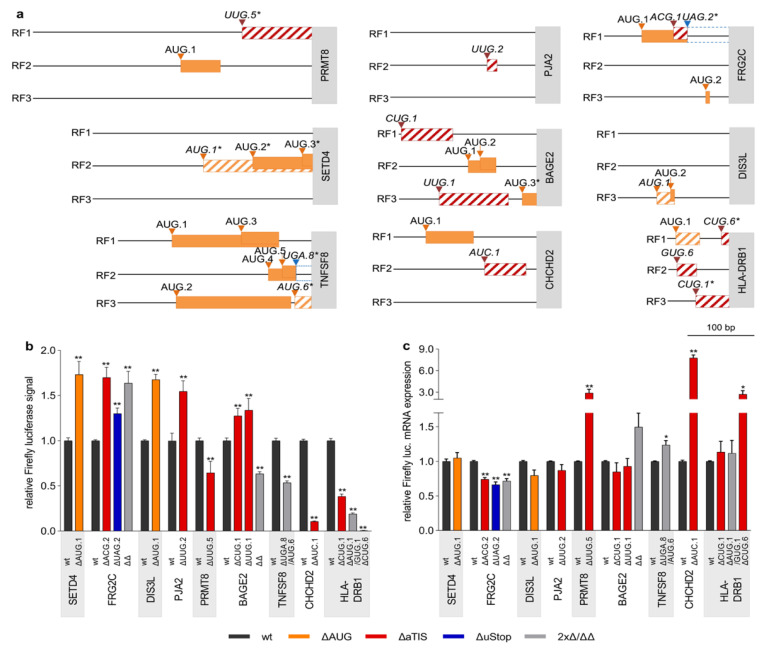
Naturally occurring cancer-associated genetic uORF variants alter downstream translational regulation. (**a**) Schematic representation of indicated TLSs, displaying the position, length and reading frame (RF, black lines) of uAUG (boxes with oblique orange lines) and aTIS (boxes with oblique red lines) uORFs affected by recurrent somatic SNVs. The start of the CDS is depicted by grey boxes containing the respective gene symbol. The lengthening of uORFs due to uStop deleting SNVs is indicated by blue hatched boxes. All AUG uORFs of respective TLSs that were not affected by genetic variation are indicated by filled orange boxes. * = original/new uStop codon is downstream of the CDS start site. (**b**) Bar graph showing the relative Firefly luciferase signals in the presence of wt TLSs (dark grey bars) and the respective ∆uAUG (orange bars), ∆aTIS (red bars), ∆uStop (blue bars) containing TLSs, normalized to Renilla luciferase signals. Results for TLSs with SNVs affecting two uORF-associated codons simultaneously and double mutant TLSs with both previously indicated SNVs (∆∆) are represented by light grey bars. (**c**) Bar graph indicating relative Firefly luciferase mRNA levels of wt uORF and ∆uORF TLSs. For each construct, Firefly luciferase mRNA levels were normalized to Renilla luciferase mRNA levels. Each bar represents data of ≥3 independent experiments, statistical analysis was performed using non-parametric Mann–Whitney-U-Test, * indicates *p* < 0.05 and ** *p* < 0.01.

The translational regulatory effects of 29 naturally occurring cancer-associated uORF variants affecting 8 uAUG, 17 aTIS, and 9 uStop codons (Table 2 and Table 3) were investigated in dual luciferase reporter assays. As individual wt TLSs differed substantially in length, structure, and the number of uAUG and aTIS codons (Supplementary Table S1), we adjusted the amounts of transfected translational control reporter plasmids to enable luciferase measurements within a similar broad linear range for each TLS (Supplementary Figure S2). Each uORF variant showed highly individual TLS contexts, including differences in the position within the TLS, the quality of the Kozak consensus sequences, and the surrounding uAUG and aTIS codons (Table 2 and Table 3, Figure 3a, Supplementary Figure S3a).

Significant alterations in relative luciferase signal were observed for 19 SNVs as compared to the respective wt TLSs, including seven ΔuAUG, nine ΔaTIS, and five ΔuStop variants (Figure 3b, Supplementary Figure S3b). Alterations of luciferase signals ranged from 1.73-fold (± 0.15, *p* < 0.01) induction for the AUG.1 > UUG variant in SET Domain Containing 4 (SETD4) to 0.006-fold (± 0.001, *p* < 0.01) repression caused by the CUG.6 > GUG variant Major Histocompatibility Complex Class II DR Beta 1 (HLA-DRB1). The SETD4 variant was observed in 1.3% of LAML patients and the HLA-DRB1 mutation occurred in 2.7%, 0.5%, and 0.3% of LAML, LUAD, and BRCA patients, respectively. To monitor transcriptional deregulation induced by the changes introduced to the TLS sequences, we concomitantly determined the mRNA expression level of Firefly and Renilla luciferases in wt and ΔuORF TLS-transfected cells (Figure 3c, Supplementary Figure S3c) and observed virtually unchanged Firefly luciferase transcript levels for the SETD4 AUG.1 > UUG TLS and an antidromic induction of HLA-DRB1 CUG.6 > GUG TLS transcript levels, implying mostly translational deregulation of luciferase activity.

Additional de-repressive effects on luciferase translation were detected for an AUG.1 > AUU variant in DIS3 Like Exosome 3′ – 5′ Exoribonuclease (DIS3L) observed in 0.9% of SKCM patients (1.68-fold ± 0.06, *p* < 0.01) and for a gain-of-AUG (ACG.1 > AUG) variant observed in FSHD Region Gene 2 Family Member C (FRG2C, 1.7-fold ± 0.11, *p* ≤ 0.01) affecting 6.1% LAML and 2.1% SKCM patients, respectively (Figure 3b). In FRG2C another somatic variant functionally deleted the corresponding uStop of the same uORF (UAG.2 > UGG) potentially generating an N-terminal extension of the FRG2C protein, associated with enhanced relative luciferase activity (1.30-fold ± 0.06, *p* ≤ 0.01). Additionally, the combination of both variants was observed in primary patient material and resulted in a similar enhancement of relative luciferase signals as the ACG.1 > AUG mutation alone (1.64-fold ±0.13, *p* ≤ 0.01, Figure 3b). Antidromic effects were observed for the ΔuORF TLS Firefly luciferase transcript levels, ranging from 0.66-fold (±0.04, *p* < 0.05) to 0.74-fold (±0.03, *p* < 0.01), suggesting that the observed induction of luciferase activity levels may underestimate the net translational effects caused by the individual genetic lesions.

In addition to the above mentioned highly repressive HLA-DRB1 CUG.6 > GUG variant we observed two other HLA-DRB1-related somatic SNVs. An HLA-DRB1 CUG.1 > CUA variant and an AUG.1 > AUA/GUG.1 > AUG were both detected in multiple types of cancer and also caused significant but somewhat less pronounced reductions of relative luciferase signals (0.39 ± 0.03, *p* < 0.01 and 0.19 ± 0.01, *p* < 0.01, respectively) as compared to the ΔCUG.6 variant (Figure 3b,c). Again, antidromic effects on the respective Firefly transcript levels for both SNVs supported the conclusion that these mutants result in strong translational but not transcriptional repression of the CDS. 

Overall, significant regulatory activity was observed for nine of 17 loss-of-aTIS and aTIS > aTIS mutations (Figure 3, Supplementary Figure S3) with additional major translational effects being observed for a UUG.2 > UUU variant in Praja Ring Finger Ubiquitin Ligase 2 (PJA2, 1.55-fold ± 0.12 *p* > 0.01), a UUG.5 > GUG variant in Protein Arginine Methyltransferase 8 (PRMT8, 0.65-fold ± 0.13 *p* < 0.01), and an AUC.1 > AUU variant in CHCHD2 (0.11-fold ± 0.01, *p* < 0.01), respectively. The somatic CUG.1 > CCG variants in BAGE Family Member 2 (BAGE2) were detected in 1.3% of LAML patients and occurred in all types of solid cancer analyzed here, with frequencies of recurrence ranging from 0.6% in PRAD to 2.1% in COAD cancer samples. This mutation was frequently observed together with another pan-solid-cancer BAGE2 aTIS variant (UUG.1 > CUG) with both variants preceding three additional uAUG codons (Figure 3a). When tested individually, both variants caused minor increases of relative luciferase activity (1.27 ± 0.08, *p* < 0.01 and 1.34 ± 0.13, *p* < 0.01, Figure 3b). Of note, the combined introduction of both variants led to a significant decrease of relative luciferase signals (0.63 ± 0.02, *p* < 0.01, Figure 3b), suggesting a complex interplay between aTIS and uAUG uORFs in the BAGE2 TLS. With respect to the observed changes in luciferase activity, antidromic mRNA levels were detected for the TLS variants of PJA2, PRMT8, CHCHD2, and BAGE2, suggesting that luciferase activity measurements underestimated the true translational effects of these variants (Figure 3c, Supplementary Figure S3c).

Another translationally repressive effect was observed for a variant simultaneously affecting a uStop and a uAUG codon in TNF Superfamily Member 8 (TNFSF8), detected in 1.3% of LAML samples. The AUG.6 > GUG variant functionally ablated a CDS overlapping uORF in RF3 and concomitantly generated novel CDS overlaps of the uAUG.4 and uAUG.5 uORFs in RF2 through a UGA.8 > UGG uStop deletion. The combined effect resulted in a marked decrease of relative luciferase activity to 0.54-fold (± 0.02, *p* < 0.01) compared to wt TLS levels, suggesting that the repressive effect of the newly overlapping uORFs was stronger than the de-repressive effect of the uAUG.6 uORF deletion. This uORF variant showed mildly induced relative mRNA expression levels of Firefly luciferase transcripts, underlining the translational effect detected in the relative luciferase assays (Figure 3c).

## 4. Discussion

Current sequencing technologies enabled the generation of large sets of whole-exome and whole-genome sequence data obtained from healthy and pathological tissue samples. While most sequencing studies focused on identifying mutations in annotated protein coding or promoter regions, the genetic variability in 5′- and 3′-regulatory sequences of human transcripts has been widely neglected [37]. Schuster and Hsieh recently summarized current knowledge about genetic variability in mRNA regulatory regions and discussed the challenges in determining the functional implication in cancer because of the incomplete understanding of TLS-mediated mechanisms [37]. In a targeted re-sequencing screen, we previously identified occasional genetic variability at uORF-associated uAUG and uStop codons in tyrosine kinases and several other human proto-oncogenes [21]. Here, we now applied an exome-wide approach and discovered multiple recurrent somatic variants in several major types of human cancer, functionally ablating uORF-associated initiation and termination codons. We focused on somatically acquired genetic lesions, because they are considered to be more relevant for cancer onset and progression as compared to germline genetic variants, as they accumulate over time and steadily increase the risk of malignant transformation [38,39]. To enhance the sensitivity of our study we considered uORF-associated variants observed in two or more patient samples to represent recurrent mutations, being aware of the fact that some of these variants may have been observed due to the background mutation rate of a particular genomic region. Currently, most computational tools correcting for these background mutation rates focus on the coding regions of genes and may therefore be of limited use in the context of TLS-associated variants [40].

Whiffin et al. recently highlighted the role of disease-associated germline variants creating or disrupting uAUG uORF initiation or termination codons, based on data collected by the Genome Aggregation Database (GnomAD) project [19]. These authors not only demonstrated that variants creating novel uAUGs or disrupting uStop codons are under strong negative selection, especially in genes intolerant to loss-of-function variants, but also provided a list of 296 genes with high-impact uORF perturbing germline variants, likely to be implicated in the pathogenesis of various diseases [19].

Extending on these findings, our current data add a significant number of cancer-associated somatic variants at uORF initiation and termination codons including aTISs, as multiple lines of evidence suggest widespread translational regulatory effects of both canonical and non-canonical uORFs [2,4,9,12,41]. Ribosome profiling demonstrated frequent translational initiation at non-AUG codons, especially within the TLSs, where near-cognate codons account for the majority of initiation events preceding the CDS [1,7,12]. Five of six SNVs exclusively deleting uAUG codons in SETD4, DIS3L, ASNS, TEDDM1, and NDST3 were found to significantly enhance downstream luciferase activity, suggesting a constitutive repressive effect on CDS translation by the majority of analyzed uAUG uORFs in wt TLS configuration. Similar repressive effects of uORFs on downstream translation have previously been observed for numerous uAUG uORFs [22,41,42] and most of these effects are readily explained by structural features of individual TLSs. For SETD4 and DIS3L, where the ΔuAUG variants caused enhanced translation of the downstream CDS, ectopic overexpression of the related proteins has previously been linked to several types of human cancer and was associated with poor overall survival [43,44]. The poorly characterized methyltransferase SETD4 was recently identified as a modulator of hematopoietic differentiation [45], suggesting that overexpression of SETD4 may also be important for the development of hematological malignancies. Similar to SETD4, functions of the exoribonuclease DIS3L are not well understood, but were roughly linked to the deregulation of the tumor-suppressor gene p53 [46]. Additionally, a knockdown of DIS3L consistently inhibited cell growth in human medulloblastoma [43].

Comparable to the translational effect of ΔuAUG uORF variants, we found eight of twelve exclusive aTIS mutations affecting AUC, ACG, CUG, GUG, and UUG start codons inducing significant changes of downstream CDS translation. Several of the uAUG and aTIS variants had repressive effects on luciferase translation, suggesting that these uORFs may serve to bypass inhibitory downstream structures of uORFs in wt TLS, as previously observed for the multi-uORF genes of yeast GCN4 and mammalian ATF4 [47,48]. Interestingly, five of twelve exclusively ∆aTIS variants did not completely ablate the initiation site, but resulted in the functional replacement of one aTIS by another. Nevertheless, four of such aTIS > aTIS variants still caused significant changes in downstream CDS translation, suggesting that individual aTIS codons may have specific functions in the TLS and are not simply replaceable by one another. Especially the CUG.6 > GUG variant in frame with the CDS start site of HLA-DRB1 almost completely abolished downstream luciferase translation. This observation may indicate that the original CUG.6 codon, but not the GUG variant, could serve as major start site of the CDS instead of the currently annotated main AUG, but this notion requires further experimental validation. HLA-DRB1 encodes for the beta chain of antigen-presenting major histocompatibility complex class II (MHCII) of HLA-DR heterodimer [49], is predominantly expressed on antigen-presenting cells and is implicated in the presentation of large processed peptides [50]. HLA-DRB1 plays a crucial role in humoral and cellular immunology, and impaired HLA-DRB1 expression was linked to the development of several diseases, including cancer [51]. Therefore, we assume that the marked reduction of HLA-DRB1 translation observed for all three uORF-associated somatic variants is likely to have (patho-) physiological consequences in vivo.

A loss of aTIS variant in the TLS of PJA2 induced significant activation of downstream luciferase translation. PJA2 is an E3 ubiquitin ligase and takes part in the inflammatory response by ubiquitylation of Malignant Fibrous Histiocytoma Amplified Sequence 1 (MFHAS1) [52], a regulator of the TLR2/NF-kB signaling pathway [53]. MFHAS1 is predicted to drive progression of colorectal cancer by integrating signals from tumor-associated macrophages [54], a cell type involved in the initiation, progression, and metastasis of several cancers [55,56]. Thus, we speculate that PJA2 overexpression in vivo may contribute to tumor progression by affecting MFHAS1 expression and function. Of note, the translation enhancing uORF variant in PJA2 occurred in four of six tumor entities investigated here, hinting towards a potentially widespread functional impact in human cancer. Four of the analyzed genes, i.e., BAGE2, FRG2C, HLA-DRB1, and NDST3, were affected by multiple recurrent somatic variants. Interestingly, eight of nine functionally tested SNVs in those genes showed significant effects on downstream translation, implying that these genes may be predominantly regulated by uORF-mediated translational control.

In our study, five of nine loss-of-uStop mutations showed regulatory effects, with the combined UGA.8 > UGG/AUG.6 > UUG mutation in TNFSF8 causing most sustained repression of luciferase reporter translation. Here, the uStop mutation lengthened the uAUG.4 and uAUG.5 uORFs in RF2, leading to a complete overlap of the uAUG.6 start site in RF3 and a new uORF overlap into the TNFSF8 CDS. Therefore, we assume that functional ablation of the uStop codon is the predominant cause for the observed decrease of CDS translation in this case. Upstream ORF-associated termination codons and the surrounding sequence context have been shown to mediate important regulatory functions, as discussed above for GCN4 [47] and recently described by Lee and colleagues [57]. In the context of carcinogenesis, a four bp frameshift mutation in a uORF of cyclin dependent kinase 1B (CDKN1B) was shown to induce a phenotype resembling multiple endocrine neoplasia syndrome type 4 by shifting the original uORF termination codon into another reading frame, leading to substantial lengthening of the uORF and the repression of CDKN1B CDS translation [29].

Overall, the dual luciferase reporter studies identified 19 functional uORF-associated variants affecting 21 codons in 16 TLSs. To demonstrate oncogenic or tumor promoting function, each uORF-associated variant described here would require independent experimental validation based on endogenous transcript variants, which was beyond the scope of the current study. Nevertheless, several lines of evidence imply that the observed alterations in uORF-mediated translational control may similarly affect endogenous protein levels of uORF-regulated genes. Apart from the abovementioned cases of disease promoting uORF defects [27,28,29], two mouse models for the CEBPB and HR genes demonstrated that translational regulation observed in reporter gene studies may often reflect endogenous mechanisms of translational control in the living organism [30,31]. For an independent set of genes, we recently stably integrated ΔuORF variants into cellular genomes by CRISPR/Cas9-mediated homology dependent repair and frequently observed similar translational regulatory effects as observed in dual luciferase reporter studies (OK and KW, unpublished data). Thus, we speculate that defective uORF-mediated translational regulation caused by the somatic mutations observed here may have contributed to the onset and/or progression of the malignant disease in at least a fraction of affected cancer patients.

## 5. Conclusions and Outlook

Our analysis revealed recurrent somatic uAUG, aTIS, or uStop mutations in a large proportion of patients suffering from six common types of human cancer. Individual uORF variants caused a wide range of activating and repressing effects on downstream translation, highlighting the need of individual experimental testing in uORF biology. We extend the catalog of translationally active uORFs by 19 somatic variants observed in patient-derived malignant tissues. The read coverage analysis of current WES datasets at uORFs underlines that available WES data still cover less than half of all potential uORF-associated initiation and termination codons, leaving room for future genome-wide analyses. Besides the uORF-mediated impact on CDS translation, recent work of others and of our group revealed that a substantial fraction of canonical and non-canonical uORF start sites serve to initiate uORF-encoded peptides [2,6,58,59]. Those uORF-peptides may form direct complexes with the associated main protein and can act in both, *cis-* and *trans*-regulatory ways. They may also sense cellular levels of small molecular regulators or metabolites serving as pepto-switches to adapt translation according to environmental signals as required [2,4]. Together with the data presented in the current study, these findings open new fields of uORF biology and warrant future investigations to decipher whether uORFs or the encoded peptides may serve as therapeutic targets for small-molecule interactors [60] to regulate translation of cancer promoting oncogenic proteins.

## Figures and Tables

**Table 1:** Summary of screened uORF-associated genomic positions, analyzed types of cancer, and identified SNVs affecting uAUG, aTIS, and uStop codons.

	uORF-Associated Nucleotides in hg38 uAUG: 190,878 aTIS: 2,515,399 uStop: 624,157						
Type of Cancer	BRCA	COAD	LUAD	PRAD	SKCM	LAML	All
Patients, *n*	1044	433	569	498	470	149	3163
	**filters to identify SNVs** ≥10 sequencing reads in tumor and normal BAM file ≥3 alternative reads in tumor VCF file SNV alters/deletes uAUG or aTIS or deletes uStop codon ≥1 TV includes the SNV						
All SNVs	7278	8756	8161	2017	26051	1913	48491
Affected patients, %	99.8	99.1	100	99.6	100	100	99.7
	**additional filters to identify somatic SNVs** ≥4 x higher alt/ref read ratio in tumor ≥2 patients with same somatic mutation						
Recurrent somatic SNVs	1029	339	494	114	3748	258	5277
Affected patients, %	68.0	67.4	68.0	38.8	86.8	75.2	66.5
Variants in ≥1% of pts.	94	37	75	14	241	258	567
Affected patients, %	35.1	40.4	31.1	20.9	61.7	75.2	38.7
SNVs w/o dbSNP entry	80	45	56	3	2112	72	2363
% of recurrent som. SNVs	7.5	13.3	11.3	2.6	56.4	27.9	44.8

**Table 2:** List of recurrent somatic SNVs affecting uAUG and aTIS codons selected for experimental analysis.

Gene Symbol RefSeq ID	Genomic Position	Codon Variant	Functional Effect	Type of Cancer (% of Patients)	uORF Size in bp	# of uAUG in TLS	uStop to CDS Distance (bp)	Kozak Context	TV with uORF
ASNS NM_001352496.2	chr7: 97928300	AUG.2 > GUG	uAUG > aTIS	BRCA (0.4)	303	4	74	++	1/7
BAGE2 NM_182482.2	chr21: 10413537	CUG.1 > CCG	loss of aTIS	COAD (2.1); LUAD (1.4); LAML (1.3); SKCM (0.9); PRAD (0.6); BRCA (1.0)	78	3	126	+	5/5
BAGE2 NM_182482.2	chr21: 10413594	UUG.1 > GUG	syn aTIS	SKCM (1.9); BRCA (1.3); PRAD (0.8); COAD (0.6)	105	3	41	−	5/5
CFH NM_000186.3	chr1: 196651949	UUG.3 > UUU/ GUG.5 > UUG	loss of aTIS/ syn aTIS	BRCA (2.2)	39/138	1	132/31	+	2/2
CHCHD2 NM_001320327.1	chr7: 56106490	AUC.1 > AUU	loss of aTIS	SKCM (8.1)	63	1	16	−	2/2
DIS3L NM_133375.4	chr15: 66294990	AUG.1 > AUU	uAUG > aTIS	SKCM (0.9)	27	2	83	++	10/13
ESRRG NM_001438.4	chr1: 216723407	GUG.1 > GGG	loss of aTIS	BRCA (3.0)	39	1	70	+	4/20
FANK1 NM_145235.5	chr10: 125896600	AGG.2 > ACG	syn aTIS	COAD (1.6)	105	0	44	+	2/3
FRG2C NM_001124759.3	chr3: 75664297	ACG.2 > AUG	aTIS > uAUG	LAML (6.0); SKCM (2.1); LUAD (0.7); PRAD (0.4)	21	3	63	+	1/1
HLA-DRB1 NM_002124.3	chr6: 32589821	AUG.1 > AUA/ GUG.1 > AUG	uAUG > aTIS/ aTIS > uAUG	LAML (1.3); LUAD (1.1); PRAD (0.6); BRCA (0.6); SKCM (0.4)	36/30	1	45/49	+	2/4
HLA-DRB1 NM_002124.3	chr6: 32589752	CUG.1 > CUC	loss of GTG	COAD (1.6); SKCM (1.3); LUAD (0.5); BRCA (0.2)	66	1	−13	+	2/4
HLA-DRB1 NM_002124.3	chr6: 32589795	CUG.6 > GUG	syn aTIS	LAML (2.7); LUAD (0.3); BRCA (0.3)	813	1	−801	−	2/4
NDST3 NM_004784.3	chr4: 118053872	AUG.4 > AUU	uAUG > aTIS	SKCM (1.1)	21	4	20	+	1/1
PJA2 NM_014819.4	chr5: 109383504	UUG.2 > UUU	loss of aTIS	LAML (2.0); LUAD (1.4); BRCA (1.3); PRAD (0.4)	15	0	58	+	1/1
PRKCQ NM_001282644.2	chr10: 6515109	UUG.2 > UUU	loss of aTIS	LAML (3.4); BRCA (2.8); LUAD (1.8); PRAD (0.6); SKCM (0.4)	156	1	−74	+	4/7
PRMT8 NM_019854.5	chr12: 3491521	UUG.5 > GUG	syn aTIS	BRCA (1.7)	1290	1	−1185	++	1/2
RBBP4 NM_001135256.1	chr1: 32651992	AUG.3 > UUG	uAUG > aTIS	LAML (2.0)	12	3	−1	−	1/3
ROPN1 NM_017578.4	chr3: 123980570	GUG.6 > GGG	loss of aTIS	BRCA (2.2); LAML (1.3)	54	4	36	−	1/3
SETD4 NM_001007261.2	chr21: 36058888	AUG.1 > UUG	uAUG > aTIS	LAML (1.3)	174	3	−8	+	4/4
SYNPR NM_144642.5	chr3: 63443357	GUG.2 > GGG	loss of aTIS	BRCA (8.2); COAD (4.4); LAML (3.4)	30	1	57	−	1/2
TEDDM1 NM_172000.4	chr1: 182400520	AUG.2 > UUG	uAUG > aTIS	LAML (2.7)	39	2	−4	+	1/1
TMIGD3 NM_001081976.2	chr1: 111563996	UUG.1 > GUG	syn aTIS	LAML (4.0); LUAD (0.7); PRAD (0.4); BRCA (0.3)	138	0	−94	+	2/3
TNFSF8 NM_001244.3	chr9: 114930326	AUG.6 > GUG	uAUG > aTIS	LAML (1.4)	147	6	−147	+	2/2

# = number; negative values in “uStop to CDS distance in bp” indicate overlap of the uORF into the CDS; The Kozak sequences surrounding the uORF initiation sites are classified as strong (++) if both core bases (underlined) match, adequate (+) if one of the core bases matches or weak (−) if none of the core bases match with the optimal Kozak consensus sequence (GCCRCCaugG).

**Table 3:** List of recurrent somatic SNVs affecting uStop codons selected for experimental analysis.

Gene Symbol RefSeq ID	Genomic Position	Codon Variant	New CDS Overlap	Type of Cancer (% of Patients)	# of uAUG in TLS	# of in-Frame uAUG + aTIS	uStop to CDS Distance in bp	New uStop to CDS Distance in bp	TV with uORF
ARPP21 NM_198399.2	chr3: 35681735	UAA.5 > UUA	yes	LAML (1.3)	3	1 + 5	15	−285	8/8
FANK1 NM_145235.5	chr10: 125896600	UAG.1 > UAC	yes	COAD (1.6)	0	0 + 3	42	−1080	2/3
FRG2C NM_001124759.3	chr3: 75664315	UAG.2 > UGG	yes	LAML (2.7); SKCM (0.6); PRAD (0.6)	3	0 + 3	63	−912	1/1
NDST3 NM_004784.3	chr4: 118053862	UAA.5 > UAU	no	LAML (2.7)	4	0 + 1	47	+24	1/1
OLIG3 NM_175747.2	chr6: 137494221	UAA.3 > AAA	no	LAML (2.7)	0	0 + 8	49	+12	1/1
ROPN1 NM_017578.4	chr3: 123980570	UGA.5 > GGA	yes	BRCA (2.2); LAML (1.3)	4	0 + 9	86	−141	1/3
TNFSF8 NM_001244.3	chr9: 114930326	UGA.8 > UGG	yes	LAML (1.3)	6	2 + 8	22	−240	2/2
ZNF596 NM_001287256.1	chr8: 240836	UAG.1 > UUG	no	LAML (3.4)	1	0 + 5	58	+51	7/7
ZSCAN21 NM_145914.3	chr7: 100056915	UAA.1 > UUA	yes	LAML (3.4)	1	1 + 0	90	−1935	4/4

# = number; negative values in “new uStop to CDS distance in bp” indicate overlap of the uORF into the CDS.

## Data Availability

The data presented in this study are available in the supplementary material.

## Supplementary Materials

The following are available online at https://www.mdpi.com/article/10.3390/biomedicines9060618/s1, Figure S1: Read coverage at uAUG, aTIS and uStop codons in five types of solid cancer and AML, Figure S2: Impact of individual wt TLS on Firefly luciferase signals, Figure S3: Naturally occurring cancer-associated genetic uORF variants alter downstream translational regulation, Table S1: Sequences of TLSs selected for experimental analysis, Table S2: List of oligonucleotides used for SDM of initiation and termination codons, Table S3: Summary of SNVs with highest rates of entity-specific recurrence, Table S4: List of recurrent somatic SNVs observed in multiple types of cancer, Data S1: Coverage analysis, Data S2: uORF associated SNVs in human cancer.
