# Supplementary material for: Somatic Functional Deletions of Upstream Open Reading Frame-Associated Initiation and Termination Codons in Human Cancer
Source: Biomedicines. 2021 May 29;9(6):618. doi: 10.3390/biomedicines9060618 (PMC8227997; doi:10.3390/biomedicines9060618)
Supplement: Supplementary file 1 [file biomedicines-09-00618-s001.zip › Juergens et al_Supplementary Figures.pdf]

Supplementary figures for

Somatic Functional Deletions of Upstream Open Reading Frame-Associated Initiation and Termination Codons in Human Cancer

Lara Jürgens, Felix Manske, Elvira Hubert, Tabea Kischka, Lea Flötotto, Oliver Klaas, Victoria Shabardina, Christoph Schliemann, Wojciech Makalowski and Klaus Wethmar

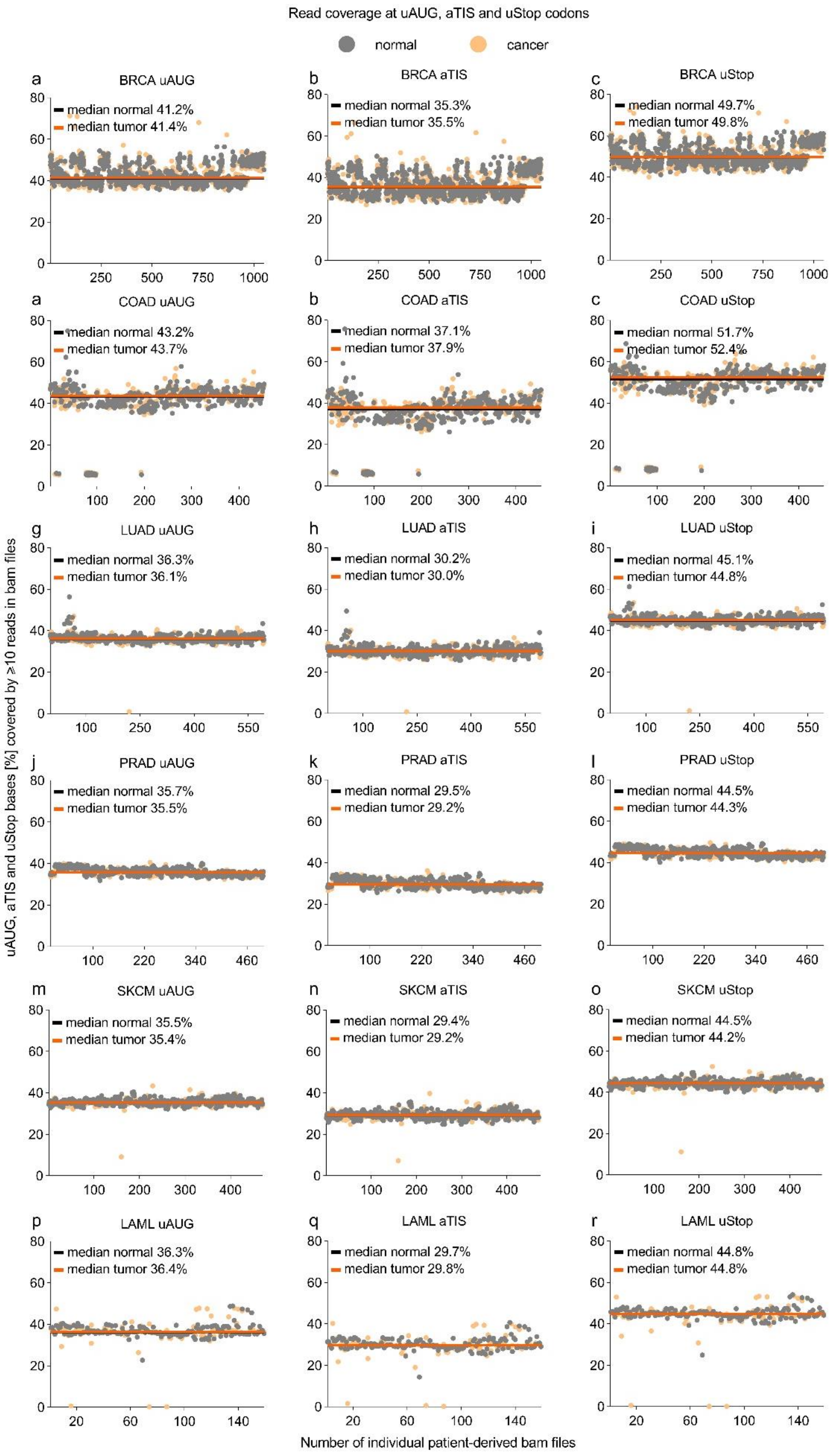

Figure S1: Read coverage at uAUG, aTIS and uStop codons in five types of solid cancer and AML

Figure legend on next page.

Figure S1: Read coverage at uAUG, aTIS and uStop codons in five types of solid cancer and AML - continued

Dot plots indicating the proportion of genomic positions of canonical (uAUG) and alternative initiation sites (aTISs), as well as the associated termination codons (uStops) covered by ≥10 reads in tumor (orange dots) and normal control (grey dots) patient samples. The coverage analysis was performed using TCGA-derived whole-exome sequencing bam files of (a-c) breast invasive carcinoma (BRCA), (d-f) colon adenocarcinoma (COAD), (g-i) lung adenocarcinoma (LUAD), (j-l) prostate adenocarcinoma (PRAD), (m-o) skin cutaneous melanoma (SKCM), (q-r) acute myeloid leukemia (LAML), and the related normal controls. The median coverage of entity-specific tumor and normal control samples is indicated by orange and black lines, respectively. Further details on the read coverage of individual samples at uAUG, aTIS, and uStop codons are given in Supplementary Data Table S1.

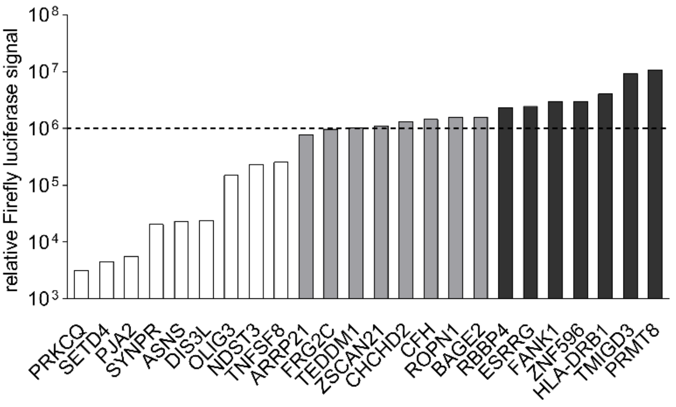

Figure S2: Impact of individual wt TLS on Firefly luciferase signals

Levels of Firefly luciferase signals (counts per second, CPS) after transfection of 10 ng wt TLS containing translational control reporter plasmid into $5x10^4$ HEK293T cells, analyzed 44h after transfection. Reporter plasmids were transfected together with 75 ng of Renilla luciferase control vector. The amount of transfected reporter plasmids were increased (white bars) or reduced (black bars) to produce wt Luciferase signals of approximately 10^6 CPS in luciferase measurements to provide a similarly wide linear range of detection for each target.

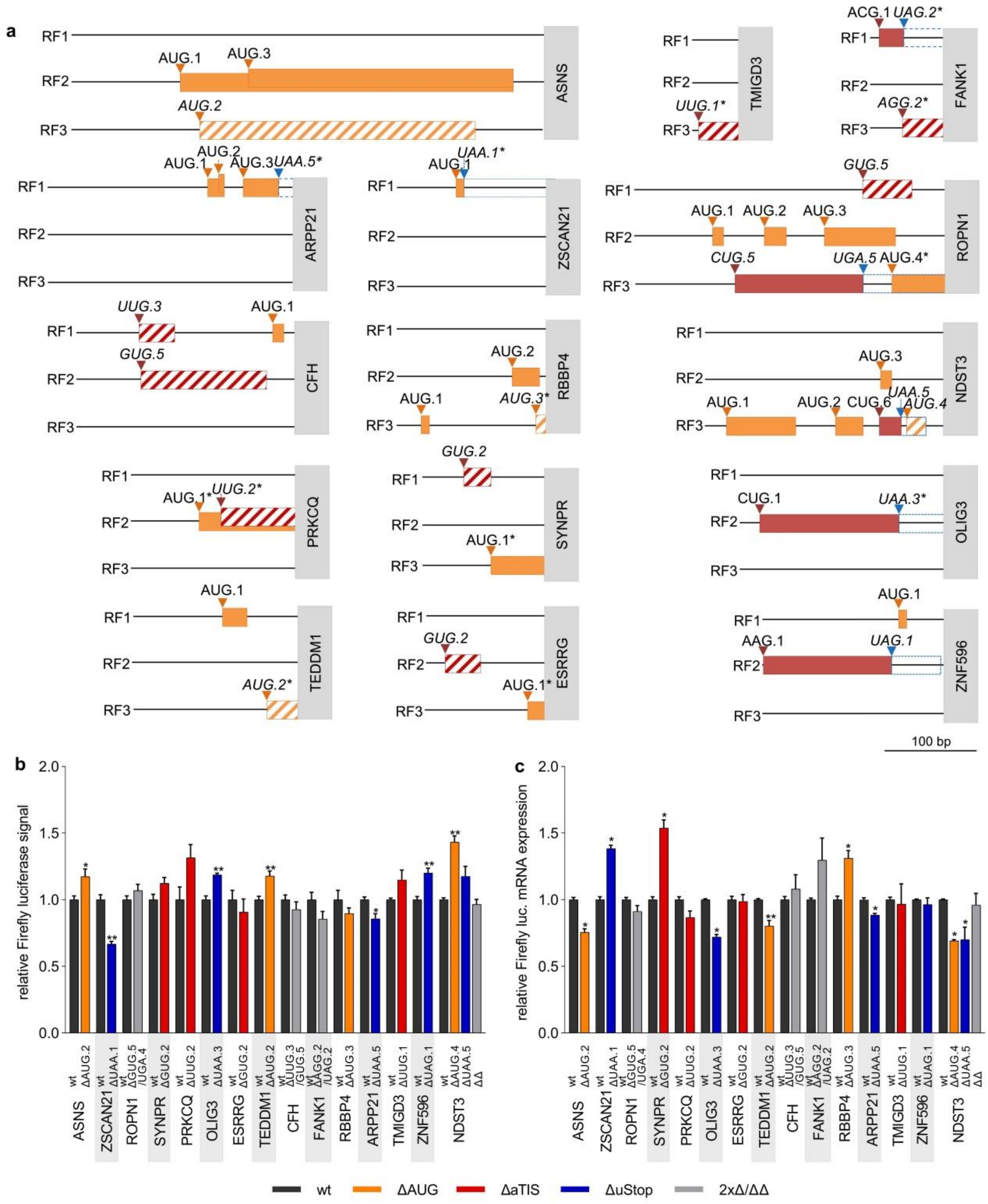

Figure S3: Naturally occurring cancer-associated genetic uORF variants alter downstream translational regulation

(a) Schematic representation of indicated TLSs, displaying the position, length and reading frame (RF, black lines) of uAUG (boxes with oblique orange lines) and aTIS (boxes with oblique red lines) uORFs affected by recurrent somatic SNVs. The start of the CDS is depicted by grey boxes containing the respective gene symbol. The lengthening of uORFs due to uStop deleting SNVs is indicated by blue hatched boxes. All AUG uORFs of respective TLSs that were not affected by genetic variation are indicated by filled orange boxes. *= original/new uStop codon is downstream of the CDS start site. (b) Bar graph showing the relative Firefly luciferase signals in the presence of wt TLSs (dark grey bars) and the respective ΔuAUG (orange bars), ΔaTIS (red bars), ΔuStop (blue bars) containing TLSs, normalized to Renilla luciferase signals. Results for TLSs with SNVs affecting two uORF-associated codons simultaneously and double mutant TLSs with both previously indicated SNVs (ΔΔ) are represented by light grey bars. (c) Bar graph indicating relative Firefly luciferase mRNA levels of wt uORF and ΔuORF TLSs. For each construct, Firefly luciferase mRNA levels were normalized to Renilla luciferase mRNA levels. Each bar

represents data of ≥3 independent experiments, statistical analysis was performed using non-parametric Mann-Whitney-U-Test, * indicates $p < 0.05$ and ** $p < 0.01$.
