# Supplementary material for: Somatic Functional Deletions of Upstream Open Reading Frame-Associated Initiation and Termination Codons in Human Cancer
Source: Biomedicines. 2021 May 29;9(6):618. doi: 10.3390/biomedicines9060618 (PMC8227997; doi:10.3390/biomedicines9060618)
Supplement: Supplementary file 1 [file biomedicines-09-00618-s001.zip › Juergens et al_Supplementary Tables.pdf]

Supplementary figures for

Somatic Functional Deletions of Upstream Open Reading Frame-Associated Initiation and Termination Codons in Human Cancer

Lara Jürgens, Felix Manske, Elvira Hubert, Tabea Kischka, Lea Flötotto, Oliver Klaas, Victoria Shabardina, Christoph Schliemann, Wojciech Makalowski and Klaus Wethmar

Table S1: Sequences of TLSs selected for experimental analysis.

TLS sequences commercially synthetized (GeneArt®, Invitrogen) for dual luciferase reporter assays. The variant codon affected by individual SNVs is underlined, written in bold letters, and displayed in wild type/mutant configuration. The CDS start codon is written in capital letters.

Gene	RefSeq ID	DNA Sequence
ARPP21	NM_198399.2	agattttaactctgaaaagccatttccagtgtctatagactattgtgagcctggagaagtagcatttagttgggatagcttcactagagctgcctgccaaagacttccttccacaggatcttgtcgcaccagcaactgacaggagcttgggagctcgggagcttgggagagggcttatgtttttaataatgtagctgtcagttcgaagcctggaaatgttgaccctcaaagggcataaaatcttgttattt**taa/tta**tttgcatctgggagaATGT
ASNS	NM_001352496.2	gcagaggagcccgcgccggccccggtgcccacctccgcgccgcgcggaccctccgagcccgcgctcagacgccccagctccgccgagaggccgcttgcgccgggtccttcttccccaaatgcaggcagagcccccggagcc**atg/gtg**gccagcccttccggcagctccaaagccactggcaagccccgaggcagggatggccggcccaggagggaggaggacgacgtccctcccgaagagaagaggctgcggctcttgctggagggggggaagcgcacagccccaggactgcgaggacggggaggacgcgccgcggccgggcagggaggagaccggcacccagacaggtggcgacggcagaggagagccaggtcggtataagcgccagcggcctcgccgcccgtcaagctgtccacatccctggcctcagcccgccacatcaccctgacctgcttacgcccagattttcttcaatcacatctgaataaatcacttgaagaaagcttatagcttcattgcaccATGT
BAGE2	NM_182482.2	gtct**ctg/ccg**gtatctcccgctgagctgctctgttcccggcttagaggaccaggagaagggggag**ttg/gtg**gaggctggagcctgtaacaccgtggctcgtctcgctctggatggtggtggcaacagagatggcagcgcagctggagtgttaggagggcggcctgagcggtaggagtggggctggagcagtaagatggcggccggagcagtaagATGG
CFH	NM_000186.4	acagcattaacatttagtgggagtgcagtgagaattgggtttaacttctggcatttctgggcttgtggc**ttgtg/ttttg**gttgatttttatttactttgcaaaagtttctgataggcggagcatctagtttcaacttccttttgcagcaagttctttcctgcactaatcacaattcttggaagaggagaactggacgttgtgaacagagttagctggtaaatgtcctcttaaaagatccaaaaaATGA
CHCHD2	NM_016139.4	ggcgttgaccgcgaaggacgaggcgtccccgtgacgtcctgctcttccaatgaacttcaggcctgcggagacgacggagaacggaagttctctgcctgtgtgctggttggttgcgcgttgaggccagccccgcctccc**atc/att**ttccggtctcctcagaagtcgcttagctcttcggtggttgtcccacgtccggaggcctagccgtcgcttacctaggATGC
DIS3L	NM_133375.5	gcccagcccttcttcacctgccgtccaggtatcatcctagtcttccctccgtctctggg**atg/att**ggaaactcttgtctagtgatgtgactcattacgtgatcccagactggaaagttgttcaagattatcttgagatccttgagtttcctgagttgaagggaattattttcATGC
ESRRG	NM_001243515.2	gagctttggtcccaactggct**gtg/ggg**cctataggcttgtcactaggagaacatttgtgttaattgcactgtgctctgtcaaggaaactttgatttatagctggggtgcacaaataatggttgccggtcgcacATGG
FANK1	NM_145235.5	aaccgtagcgacgccggccctggctgagaggcgt**tagg/tacg**agtccgggggttcgcccgcggaggccggggagcagccgaccATGG
FRG2C	NM_001124759.5	gtccccagtagaactacagcaagaaacttctgattgaggctctaagaagcggcaggaatgagaaaactcttcagccaataagagtaagccacgcccagccgaggg**acg/atg**tataaaaggcaggtc**tag/tgg**cagactaacccacactctgcctttggacatgtgagagagcgcacctttcacttgagcttcaacATGG
HLA-DRB1	NM_002124.4	cctataacttgga**atgtg/atatg**ggtggaggggttcatagttctcc**ctg/ctc**agtgagacttgcctgcttctctggcccctggtcctgtc**ctg/gtg**ttctccagcATGG
NDST3	NM_ 004784.3	gtaaagtcaaaaagcctgagccgatggtaacgagtggtactgtttccgtttcttttccatctattgcaactttctcaagcattttcagctctgaactttaattccagactgtattttctgtgagtcctgatcaagtgatacaaatgagctgcaatggtgacataaactcttgacagagattggaaaagtagctggaacaccatcttttcttt**taa/tat**cttttt**atg/att**gtgcttctgttggcatagttggggaaagcacctacaacATGA
OLIG3	NM_175747.2	agttcaagcacacaccaactagctgtccaggaagcagcctaaccagagagaggggcgtgggagggagagaggcggggaaaagcagcgtctccgcttttcttctcacgtcttgccgcggagaagtgggttcctgggcagtgcagatttttttttttaattcaagattttctt**taa/aaa**tttcccgcctaaagcctccccacggtagggccgcatcccctgtaataaaATGA
PJA2	NM_014819.5	accgccgccgccgccgccacagccgccagccgcttcgcgttcggcgggggaggagttggaggcggagaagaaggcggtggtggcgggtggaggttcgagcgctgttctcgctccggagccgctgcacatttcggaatcttctgcggc**ttg/ttt**tccatagtgtgaataaaaactaaatcacatctataattctactgaactggtcacacagacgctgccatatATGT
PRKCQ	NM_001323266.2	agtccccgcgcagtccccgcgcagtcccagcgccaccgggcagcagcggcgccgtgctcgctccagggcgcaaccatgtcgccatttcttcggattggc**ttg/ttt**tccaactttgactgcgggtcctgccagtcttgtcagggcgaggctgttaacccttactgtgctgtgctcgtcaaagagtATGT
PRMT8	NM_019854.5	gcttcgcccagcggatcggcagaagttgagaggagttggcggctgcctccggccggccggactttgcgagcagcctggagaggatccgcgaccgccgccgccgccgcggaggcttcggggctgcttccctcgagcttagcccgcagcgcgggtggagaggggcggggagggggtcgggggcacgagaagaacttgaaaccgtgtgaaggaatccggagcagatgagaagggaggaaaataaaagaaagtggagactgcagaacagactccgctgtggctgactgtgccggccgacgctccagctgaggggctggg**ttg/gtg**gattttttttttctcccatcctctcgctctctcttttaaagcgacaccagctctctcctcctctactatctcggtatcaccaaacccttgccggctcttATGG
RBBP4	NM_001135256.2	gtgcctgggctgagccgcggcctcgacatggcctaacagtgagggcaggcccgagtttgccgagtgcagtccccgcagccttcgacgacgcagtggaagaacgagtgatcaacgaggaatacaaaatatggaaaaagaacaccccttttcttt**atg/ttg**atttggtgATGA
ROPN1	NM_017578.4	tagcacttcatagactgctatcacgcattcatttttactagtacctattgaacactaagttccaggcactgggctaggcactgggatggtgtggtgagcaaaaactgaccagtcctgccgtggagtttgctgggggagacacatgttactcaaagaatcacactaaggatagcaatttatctcaaagctgcaagttcctgccatctacatgtgcccagagtccccaaaattgagtgtcaaacagaggctgg**gtga/ggga**cctcctgtccaggattgctctcatcgtatgaatcgaagttttcatcttaacgtatttttttctcctgagaataggccaaccaatcaATGG
SETD4	NM_001286752.2	gggagactgaggacttggattgcttaaggacaccaggcaggcgcgacagccttcagcctctccctctgcagttttggccggttcctttaacttgccttcatctggggctgtttggtatttccacgtgccttgggcccgcccactgcagcctccatcttggaagcggccgccggcgcctagattgagcatttccacagaaattacagttttgtcctttttgaaaaaatagaactgtatttcagaaaaaagaaactacagttttagc**atg/ttg**cagaaaggaaaagggagaacaagccggatcagaagacgaaaactctgcggaagttctgaatcaagaggagagatggggtctttctgtgttgggcaggctgggctcaaactcctggcctcaagcagtcctcccgccttggcctcccaaatgctgggaatacagaATGA
SYNPR	NM_144642.5	cttcttctcctcctttgcttcataaaaagagggacaagtggctg**gtg/ggg**ctgtggacagagaagctttatttttagtatgagacaacctctattttctttcaggagagggaagttggattatcaattcttttgtaaATGT
TEDDM1	NM_172000.4	atatataggcccagaactcgagaggcctgtgagggagaggagagcagagaggtcctgagtattgctcttcacgcttcagtcggacaggcctgtattccatgggaaagtccattggccatttttaactggggctatttcttttctctt**atg/ttg**gactatatcaggccacagtggtctccaagggcATGA
TMIGD3	NM_001081976.2	gtggctg**ttg/gtg**gaagcttctgtggcagttggtagagaccaccagttggaccaATGG
TNFSF8	NM_001252290.1	aaccgtcattttcctacgcgccctctgacatcagccaccttctctgtagctagtttctctgcacacaacttaatccctggcaatgaaaaatgaacctctcccccacccttgctgccgcctctcgcctcacgcccccagagaagagtttctccaccaggcagcaggtgaaggtttttttccaagtcacatgattcaggattcagggggagaatccttcttggaacagagatgggcccagaactgaatcagatgaagagagataaggtg**tgatg/tggtg**tggggaagactatataaagaATGG
ZNF596	NM_001287256.1	aagttgggtggaagcagcgcggacccacggcacaccgaacgcactccaacagaacccgacgcagacacgcgctttcaaccggcggagacactggcagggccagaaacgcgcgcagcgggggcgggagatttgtcttct**tag/ttg**tgcttggatggtgtgagtgaaaacccagaggaatacatttggtggctgagctagtacaATGC
ZSCAN21	NM_001362781.2	acttccgggtgcgcggtcccgaggtacgcggtgcggtctcccggtacccggagcggctctgattcatggttt**taa/tta**cttgtggccctaaagaactggaaacccaaaggaacgaatattcctgccccacagagtcccatctttggtgaggctgtttctggagtttacATGA

Table S2: List of oligonucleotides used for SDM of initiation and termination codons.

All forward and reverse oligonucleotides (primers) used for site directed mutagenesis (SDM) are given in 5′-3′-sequence direction. The variant codons are written in bold capital letters, with mutated base underlined.

Induced SNV	RefSeq_ID	forward primer	reverse primer
BAGE2 ΔCUG.1	NM_182482.2	gcccggggtct**CCG**gtatctcccgc	gcgggagatac**CGG**agaccccgggc
BAGE2 ΔUUG.1	NM_182482.2	gagaagggggag**CTG**gaggctggag	ctccagcctc**CAG**ctcccccttctc
FANK1 ΔAGG.2/UAG.1	NM_145235.5	ctgagaggcgt**TACG**agtccgggggttc	gaacccccggact**CGTA**acgcctctcag
FRG2C ΔACG.2	NM_001124759.5	cccagccgaggg**ATG**tataaaaggcag	ctgccttttata**CAT**ccctcggctggg
FRG2C ΔUAG.2	NM_001124759.5	gtataaaaggcaggtc**TGG**cagactaacccacac	gtgtgggttagtctg**CCA**gacctgccttttatac
HLA-DRB1 ΔAUG.1/GUG.1	NM_002124.4	ctataacttgga**ATATG**ggtggaggggttc	gaacccctccacc**CATAT**tccaagttatag
HLA-DRB1 ΔCUG.1	NM_002124.4	ggttcatagttctcc**CTC**agtgagacttgc	gcaagtctcact**GAG**ggagaactatgaacc
HLA-DRB1 ΔCUG.6	NM_002124.4	ctggtcctgtc**GTG**ttctccagcatg	catgctggagaa**CAC**gacaggaccag
NDST3 ΔAUG.4	NM_ 004784.3	cttttt**ATT**gtgcttctgttggc	gccaacagaagcac**AAT**aaaaag
NDST3 ΔUAA.5	NM_ 004784.3	caccatcttttcttt**TAT**ctttttatggtg	caccataaaaag**ATA**aaagaaaagatggtg
TMIGD3 ΔUUG.1	NM_001081976.2	ctagcgtggctg**GTG**gaagcttctg	cagaagcttc**CAC**cagccacgctag

Table S3: Summary of SNVs with highest rates of entity-specific recurrence

# = number. The average dbSNP frequency was calculated using individual frequencies according to the TopMed, ExAC, and GnomAD projects. If none of those contained variation data for individual SNVs “NONE” is displayed.

entity	most frequent uORF positions w/o dbSNP annotation				most frequent uORF positions with dbSNP annotation				
	gene	genomic position	effect	affected patients (%)	gene	genomic position	effect	affected patients (%)	dbSNP ID (average frequency, %)
BRCA	ROPN1	chr3:123980570	GUG>GGG; UGA>GGA	2.2	MELK	chr9: 36599396	UAA>GAA	8.6	rs374077047 (3.5)
	RABL2A	chr2:113628561	AUA>AUC	2.2					
COAD	OR2H1	chr6:29461656	AAG>GAG	1.6	SYNPR	chr3: 63443357	GUG>GGG	4.4	rs1183102673 (NONE)
LUAD	CBWD2	chr2:113437882	UUG>GUG	0.9	NBPF20	chr1: 145405250	UUG>UUU	3.3	rs1412188415 (0.01)
	POM121	chr7:72890655	GUG>GGG	0.9	ZNF7	chr8: 144829396	CUG>AUG	3.3	rs774406977 (0.1)
	MRGPRX4	chr11:18173175	GUG>GGG	0.9					
PRAD	VEZF1	chr17:57983388	UGA>AGA	2.0	CDC27	chr17: 47156984	AGG>AAG	4.6	rs772436784 (0.9)
SKCM	HMGXB4	chr22:35262339	ACG>ACU	2.5	CHCHD2	chr7: 56106490	AUC>AUU	8.1	rs867193277 (0.0008)
LAML	FDCSP	chr4:70234185	UAA>AAA	6.0	NBPF20	chr1: 145405250	UUG>UUU	10.1	rs1412188415 (0.01)

Table S4: List of recurrent somatic SNVs observed in multiple types of cancer

The average dbSNP frequency was calculated using individual frequencies according to the TopMed, ExAC, and GnomAD projects. If none of those contained variation data for individual SNVs "NONE" is displayed.

gene symbol ID	genomic position	variant	effect	entity (% of patients with somatic mutation)	dbSNP ID (average frequency, %)
AQP12B	chr2: 240682856	AUC>GUC	loss of aTIS	COAD (0.9); LUAD (0.9); PRAD (0.6); SKCM (0.6); BRCA (0.2)	rs760345459 (0.02)
AQP12B	chr2: 240682857	AUC>UUC	loss of aTIS	LUAD (0.9); COAD (0.7); PRAD (0.6); SKCM (0.4); BRCA (0.2)	rs766089521 (0.02)
BAGE; BAGE5; BAGE4	chr21: 10413594	UUG > CUG	aTIS>aTIS	SKCM (1.9); LUAD (1.6); BRCA (1.3); PRAD (0.8); COAD (0.7)	rs75318310 (NONE)
HLA-DRB1	chr6: 32589772	CUG > GUG	aTIS>aTIS	LUAD (0.5); COAD (0.5); BRCA (0.5); SKCM (0.4); PRAD (0.4)	rs17204744 (0.003)
TAS2R43	chr12: 11092279	AUU>ACU	loss of aTIS	COAD (0.9); BRCA (0.6); LUAD (0.5); SKCM (0.4); PRAD (0.4);	rs369719862 (0.3)
BAGE; BAGE5; BAGE4	chr21: 10413537	CUG>CCG	loss of aTIS	COAD (2.0); LUAD (1.4); LAML (1.3); BRCA (1.0); SKCM (0.9); PRAD (0.6)	rs78230864 (NONE)
CDC27	chr17: 47156984	AGG>AAG	aTIS>aTIS	PRAD (4.6); SKCM (2.3); LUAD (1.9); LAML (1.3); BRCA (1.1); COAD (0.7)	rs772436784 (0.9)
GRM5	chr11: 89047888	UGA>UUA; UUG>UUU	loss of uStop, loss of aTIS	BRCA (3.4); LAML (2.0); LUAD (1.6); COAD (0.9); SKCM (0.4); PRAD (0.4)	rs200235727 (1.2)
HLA-DRB5	chr6: 32530234	CUG>CUC	loss of aTIS	LAML (3.3); SKCM (2.3); BRCA (1.4); PRAD (1.0); LUAD (0.9); COAD (0.9)	rs3179203 (1.6)
LYZL2	chr10: 30629668	AAG>AGG; UAA>UAG	aTIS>aTIS; uStop>uStop	COAD (3.2); SKCM (2.8); LAML (2.0); PRAD (2.0); BRCA (1.8); LUAD (1.1)	rs74551644 (29.3)
LYZL2	chr10: 30629732	UUG>UUA	loss of aTIS	PRAD (2.8); SKCM (2.8); COAD (2.5); BRCA (2.5); LAML (2.0); LUAD (1.9)	rs777979230 (0.08)
MSANTD2	chr11: 124774825	UUG>UUU	loss of aTIS	LUAD (3.0); BRCA (2.3); LAML (2.0); PRAD (1.4); SKCM (0.9); COAD (0.5)	rs75374326 (6.6)
PRSS2	chr7: 142770963	AUC>GUC	loss of aTIS	COAD (2.7); LAML (2.7); BRCA (1.8); LUAD (0.9); SKCM (0.6); PRAD (0.4)	rs771586111 (NONE)
ST6GALNAC4	chr9: 127916493	GUG>GGG	loss of aTIS	BRCA (5.2); LUAD (1.6); LAML (1.3); COAD (1.2); PRAD (0.8); SKCM (0.6)	rs375089890 (NONE)
TAS2R43	chr12: 11092250	AUU>UUU	loss of aTIS	LAML (2.0); LUAD (1.4); PRAD (1.4); COAD (1.2); BRCA (1.2); SKCM (0.6)	rs779232783 (0.3)
TAS2R43	chr12: 11092256	UAA>UUA	loss of uStop	LAML (2.0); PRAD (1.4); LUAD (1.4); BRCA (1.2); COAD (1.2); SKCM (0.6)	rs748363493 (0.3)
TRMT1L	chr1: 185150477	UUG>UUU	loss of aTIS	LUAD (2.8); LAML (2.0); BRCA (1.4); SKCM (1.3); PRAD (1.2); COAD (0.5)	rs78652979 (4.8)
