# Supplementary figures and images for: Somatic Functional Deletions of Upstream Open Reading Frame-Associated Initiation and Termination Codons in Human Cancer
Source: Biomedicines. 2021 May 29;9(6):618. doi: 10.3390/biomedicines9060618 (PMC8227997; doi:10.3390/biomedicines9060618)

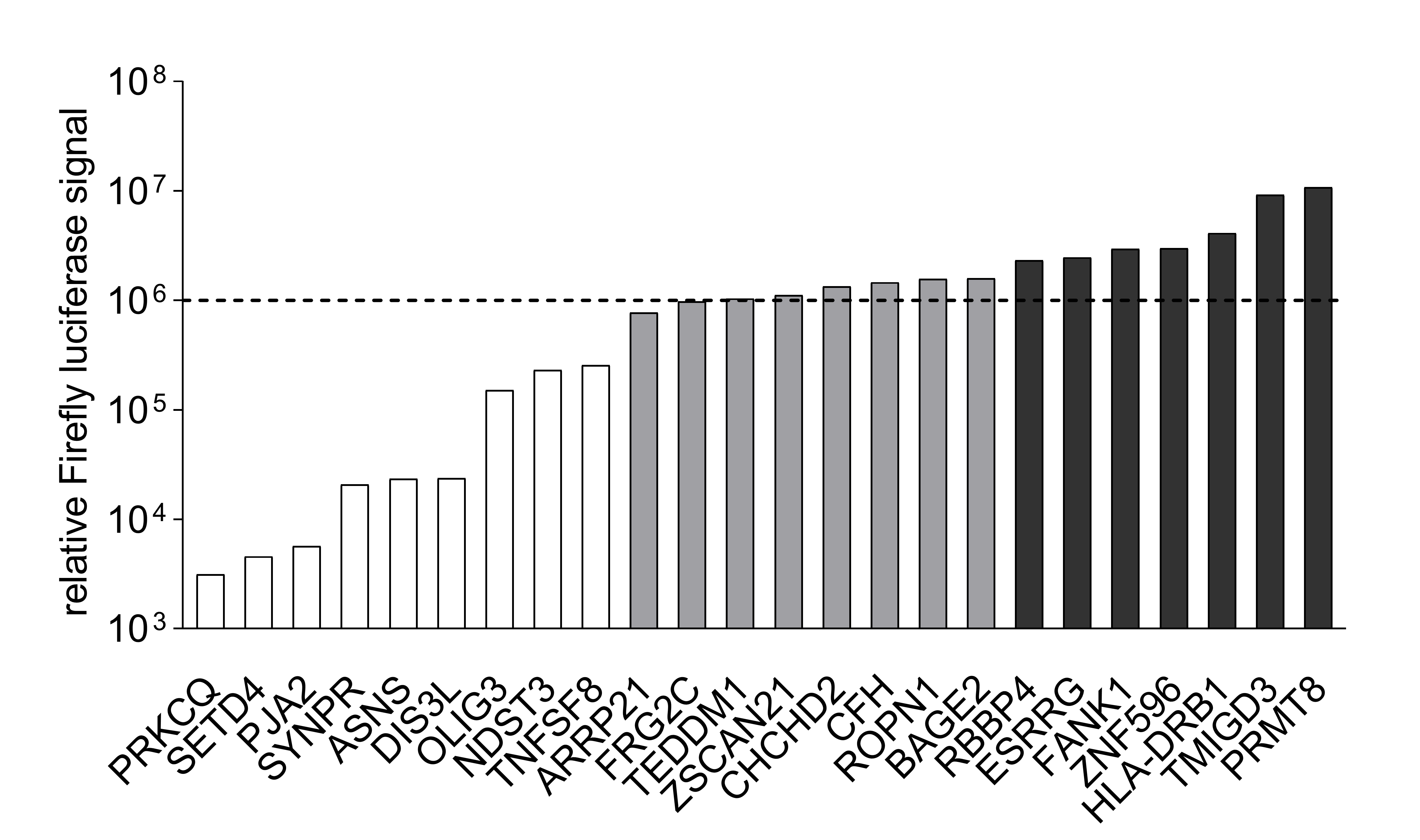

Supplement: Supplementary file 1 [file biomedicines-09-00618-s001.zip › Supplementary Figure S2_proofread.tif]
